# The current application of the Royston-Parmar model for prognostic modeling in health research: a scoping review

**DOI:** 10.1186/s41512-018-0026-5

**Published:** 2018-02-07

**Authors:** Ryan Ng, Kathy Kornas, Rinku Sutradhar, Walter P. Wodchis, Laura C. Rosella

**Affiliations:** 1 Dalla Lana School of Public Health, University of Toronto, 155 College St, Toronto, ON M5T 3M7 Canada; 2 Institute for Clinical Evaluative Sciences, 2075 Bayview Ave, Toronto, ON M4N 3M5 Canada; 3 Institute of Health Policy, Management and Evaluation, University of Toronto, 155 College Street, Toronto, ON M5T 3M6 Canada

**Keywords:** Survival analysis, Flexible parametric survival models, Royston-Parmar models, Prediction modeling, Scoping review

## Abstract

**Background:**

Prognostic models incorporating survival analysis predict the risk (i.e., probability) of experiencing a future event over a specific time period. In 2002, Royston and Parmar described a type of flexible parametric survival model called the Royston-Parmar model in *Statistics in Medicine*, a model which fits a restricted cubic spline to flexibly model the baseline log cumulative hazard on the proportional hazards scale. This feature permits absolute measures of effect (e.g., hazard rates) to be estimated at all time points, an important feature when using the model. The Royston-Parmar model can also incorporate time-dependent effects and be used on different scales (e.g., proportional odds, probit). These features make the Royston-Parmar model attractive for prediction, yet their current uptake for prognostic modeling is unknown. Thus, the objectives were to conduct a scoping review of how the Royston-Parmar model has been applied to prognostic models in health research, to raise awareness of the model, to identify gaps in current reporting, and to offer model building considerations and reporting suggestions for other researchers.

**Methods:**

Five electronic databases and gray literature indexed in web sources from 2001 to 2016 were searched to identify articles for inclusion in the scoping review. Two reviewers independently screened 1429 articles, and after applying exclusion criteria through a two-step screening process, data from 12 studies were abstracted.

**Results:**

Since 2001, only 12 studies were identified that used the Royston-Parmar model in some capacity for prognostic modeling, 10 of which used the model as the basis for their prognostic model. The restricted cubic spline varied across studies in the number of interior knots (range 1 to 6), and only three studies reported knot placement. Three studies provided details about the baseline function, with two studies using a figure and the third providing coefficients. However, no studies provided adequate information on their restricted cubic spline to permit others to validate or completely use the model.

**Conclusions:**

Despite the advantages of the Royston-Parmar model for prognostic models, they are not widely used in health research. Better reporting of details about the restricted cubic spline is needed, so the prognostic model can be used and validated by others.

**Registration:**

The protocol was registered with Open Science Framework (https://osf.io/r3232/).

**Electronic supplementary material:**

The online version of this article (10.1186/s41512-018-0026-5) contains supplementary material, which is available to authorized users.

## Background

Prediction models are used in health research to relate pieces of information (i.e., predictors) to identify the probability of a state (i.e., outcome), such as whether a specific disease or condition currently exists, which is known as diagnosis, or the probability the state will occur in the future, which is known as prognosis [1, 2]. In health research, prognostic models are used for predicting the risk of the future event (i.e., probability), such as the onset of disease (i.e., incidence), disease progression or mortality over a specified time period in individuals, or subgroups of the population [1]. While prognostic models can be constructed using various techniques (e.g., neural networks, classification trees), regression modeling is the most commonly used technique to develop, validate, and update prediction models in health research [3]. When regression modeling is used for prognostic models, survival analysis is a common regression model type because it accounts for the relationship between the predictor(s) and the outcome(s) and the time until the occurrence of the outcome(s) [4, 5]. While predictions are often presented for a specific time point, prognostic models based on survival analysis enable predictions to be a function of study follow-up time, which increases the application of the model for different contexts. Examples of prognostic models that use regression modeling for individual risk prediction are the Framingham risk score and the Gail model [6, 7], which measure the future risk of cardiovascular disease and breast cancer development, respectively. Prognostic models can also be used for predicting the risk of disease at the population level [8], one example being a tool that predicts the population risk for diabetes to aid health services planning and delivery [9].

For some prognostic models, the framework for survival analysis is based on the Cox proportional hazards (PH) model [10]. First described in 1972 [11], the Cox PH model is widely used due to its ease of calculating the relative effects of hazards between groups (i.e., hazard ratio (HR)) without needing to estimate the baseline hazard function. The Cox PH model treats the baseline hazard function as a nuisance parameter, so the partial likelihood function is maximized, which permits estimation of the regression parameters, but not the baseline hazard function. As a result, absolute measures of effects (e.g., survival probability, hazard rates) can only be estimated at the event times, which results in a step function where the estimate at one event is held constant and carried forward until the time of the next event. Two common semi-parametric methods to estimate the survival function post hoc from the Cox PH model are the Breslow estimator, which is a generalization of the Nelson-Aalen estimator of the cumulative hazard [12], and the Kalbfleisch-Prentice estimator, which is an extension of the Kaplan-Meier estimator of survival [13]. The distance between steps can be reduced by using data with large sample sizes and many observed events across the study period, which permits risk prediction at many time points. Another solution is to use a smoothing method (e.g., locally weighted smoothing) on the survival curves. However, the optimal approach for prognostic models would be to utilize the baseline hazard function for the continuous mathematical estimation of the absolute measures of effect.

Parametric survival models permit direct estimation of absolute measures of effect because an underlying distribution is specified mathematically. Parametric survival models specify the baseline hazard (*h*_0_(*t*)), and a common specification is the Weibull distribution, which is a function of a scale parameter (*λ*), a shape parameter (*γ*), and time (*t*), defined as:

1 $$ {h}_0(t)=\lambda \gamma {t}^{\gamma -1}, $$

where the scale and shape parameters must both be greater than 0. The shape parameter defines the shape of the Weibull function, which generally is a monotonically increasing (*γ* > 1) or decreasing (*γ* < 1) hazard. The exception is when the shape parameter equals one (*γ* = 1), in which case the baseline hazard is reduced to an exponential distribution with a constant hazard. In situations where the underlying hazard is monotonically increasing, monotonically decreasing, or constant, the Weibull distribution can provide accurate predictions for absolute measures of effect [9]. However, when the hazard function has a more complex shape (e.g., bathtub shape, J shape), specifying a Weibull distribution will lead to inaccurate predictions and/or model convergence issues.

In 2002, Royston and Parmar published a paper in *Statistics in Medicine* describing the Royston-Parmar model, a type of flexible parametric survival model [14]. This model features a restricted cubic spline, which solves issues encountered when using the Cox PH and parametric PH survival models. The restricted cubic spline permits estimation of a continuous function instead of a step function. As well, more complex shapes can be modeled, which avoid inaccurate predictions and model convergence issues. In the PH context, the Royston-Parmar model can be thought of as a generalization of the Weibull distribution where a general function for the baseline log cumulative hazard function on the log timescale is modeled instead of a linear function (as is the case when a Weibull distribution is pre-specified). The log cumulative hazard function on the log timescale for a Weibull distribution is:

2 $$ \ln \left(H(t)|{x}_{\mathrm{i}}\right)=\ln \left(\lambda \right)+\gamma \ln (t)+\boldsymbol{\beta} {\boldsymbol{x}}_{\mathbf{i}}, $$

where the first two terms—ln(*λ*) and *γ* ln(*t*)—describe the linear baseline function with respect to log time, and the third term—***βx***_**i**_—represents a vector of covariates, each weighted by a coefficient. This linear baseline function could be generalized and re-written as:

3 $$ \ln \left(H(t)|{x}_{\mathrm{i}}\right)=\ln \left[{H}_0(t)\right]+\boldsymbol{\beta} {\boldsymbol{x}}_{\mathbf{i}}, $$

where ln[*H*_0_(*t*)] represents a general baseline log cumulative hazard function. Royston and Parmar proposed to model the general baseline log cumulative hazard function on the log timescale as a restricted cubic spline. A spline is a piecewise function in which the boundaries of each sub-function are defined by knots. The knots at either end of the spline are called boundary knots, and all knots between the boundary knots are called interior (aka internal) knots. Constraints are used to ensure the sub-functions are connected at the knots in a smoothed fashion so that the spline function, its first derivative, and its second derivative are continuous. A cubic spline is a spline in which all sub-functions are cubic curves. A restricted cubic spline (aka natural cubic spline) is a cubic spline with an additional restriction where the first and last sub-functions beyond the boundary knots are linear functions instead of cubic functions. A restricted cubic spline can be expressed as [15]:

4 $$ s(x)={\eta}_0+{\eta}_1{z}_1+{\eta}_2{z}_2+\dots +{\eta}_{K-1}{z}_{K-1}K $$

where *K* is the number of knots, *z*_i_ are derived variables, and *η*_i_ are the coefficients for the derived variables. The derived variables can be calculated as:

5 $$ {z}_1=x $$

6 $$ {z}_{\mathrm{j}}={\left(x-{k}_{\mathrm{j}}\right)}_{+}^3-{\phi}_{\mathrm{j}}{\left(x-{k}_1\right)}_{+}^3-\left(1-{\phi}_{\mathrm{j}}\right){\left(x-{k}_{\mathrm{j}}\right)}_{+}^3\ \mathrm{for}\ j=2,\dots, K-1. $$

where *k*_i_ represents the position of the *i*th knot and *ϕ*_j_ = (*k*_K_ − *k*_j_)/(*k*_K_ − *k*_1_). It is possible for the derived variables to be correlated, so the derived variables can be orthogonalized using the Gram-Schmidt orthogonalization [16]. When the intercept is not considered, the degrees of freedom are equal to the number of interior knots plus one. The Royston-Parmar model under a PH context can be expressed mathematically as:

7 $$ \ln \left(H(t)|{x}_{\mathrm{i}}\right)=s\left(\ln (t)|\eta, {k}_0\right)+\boldsymbol{\beta} {\boldsymbol{x}}_{\mathbf{i}}. $$

where *s*(ln(*t*)| *η*, *k*_0_) is a restricted cubic spline that is a function of the coefficients of the derived variables (*η*) and the number of knots (*k*_0_) (Eq. 4). The restricted cubic spline permits baseline log cumulative hazard functions with complex shapes to be fit, including functions with multiple increasing and/or decreasing regions. The boundary knots are placed at the extreme uncensored survival times, which improve the stability of the fitted function [14, 17]. Specifying the number and placement of the interior knots defines the shape of the baseline log cumulative hazard function. More interior knots increase the flexibility of the fitted function at the risk of overfitting the model; conversely, fewer interior knots decrease the flexibility of the fitted function at the risk of underfitting the model. When no interior knots are specified, the restricted cubic spline reduces to a linear function (i.e., the Weibull distribution). Knot placement is also important. Interior knots spaced further apart will capture the overall shape of the function at the risk of missing the nuances of the function; conversely, interior knots spaced closer together will capture more nuances at the risk of modeling random noise. While previous work has shown the number and placement of knots to be robust for modeling the baseline function [14, 18–20], varying the number and placement of knots in a sensitivity analysis helps ensure the baseline function is well specified. Sensitivity analysis can also be performed to examine the fit of the restricted cubic spline on other scales (i.e., the proportional odds (PO) scale as an extension for the log-logistic distribution, or the probit scale as an extension for the log-normal distribution) because the Royston-Parmar model was developed within the Aranda-Ordaz family of link functions [14]. Since 2002, Royston-Parmar models have been extended to relative survival models in 2007 [19]. In the context of mortality, relative survival models compare the observed all-cause survival for a group of individuals (e.g., cancer patients) (*S*(*t*)) relative to the expected survival of a comparable group representing the general population (S*(*t*)) to obtain an estimate of relative survival (*R*(*t*)). The corresponding hazard function describes the excess hazard rate associated with the group of interest (*δ*(*t*)) as the difference between the observed hazard rate for the group of interest (*h*(*t*)) and the expected hazard for the general population (h*(*t*)).

8 $$ R(t)=\frac{S(t)}{S^{\ast }(t)} $$

9 $$ \delta (t)=h(t)-{h}^{\ast }(t) $$

Relative survival indicates how much worse (or better) the survival is for the group of interest relative to the general population and is used when the cause of death is inaccurate or unknown. Recently, the Royston-Parmar model has been extended to the cure model (2011) [17, 21], a relative survival model where the excess hazard becomes zero over time (*δ*(*t*) = 0) for individuals who are still alive; cause-specific competing risks where individuals are at-risk for more than one outcome, and the occurrence of one outcome prevents or alters the probability of another outcome occurring (2013) [22]; and joint modeling of longitudinal and survival data (2011) [23].

In the PH context, Royston-Parmar model can be thought of as a hybrid approach of the parametric survival model and the Cox PH model. Modeling the baseline log cumulative hazard function as a restricted cubic spline is similar to the parametric survival model, but the complexities of the baseline function can be modeled without sacrificing model fit. The restricted cubic spline can also be reported mathematically as a function of derived variables and their coefficients and the number of knots (Eq. 4). This expression permits the continuous estimation absolute and relative measures of effect and their uncertainty, which is an advantage versus the Cox PH model combined with an estimator. Continuous estimation of absolute measures of effect (e.g., hazard rates, differences in survival probability, standardized survival function, population-averaged survival function) increases the applicability the prognostic model because time-specific predictions can be made by the user. Despite these advantages of the Royston-Parmar over the Cox PH model (with an estimator) and parametric survival models for prognostic modeling, the use of the model in health research for prognostic models is unknown. Thus, a scoping review of the application of the Royston-Parmar model for prognostic models in health research was conducted to document its current use, to raise awareness of the model, to identify gaps in current reporting, and to offer recommendations for future reporting.

## Methods

### Scoping review framework

To achieve our objective of documenting the current use of the Royston-Parmar model for prognostic models in health research, we chose the scoping review framework proposed by Arksey and O’Malley and refined by the Joanna Briggs Institute [24, 25]. A scoping review is a knowledge synthesis strategy that provides a broad overview of a topic by mapping the breadth and depth of its evidence. Like a systematic review, a scoping review uses a systematic, predefined search strategy to comprehensively search the literature; however, scoping reviews seldom critically appraise the quality of the literature using a scoring instrument or include a meta-analysis.

### Study question

The study question follows the Population, Concept and Context elements which have a broader scope and aligns better with a scoping review than the traditional Population, Intervention, Comparison, Outcome elements used for systematic reviews [25]. The specific question of this study was “To date, how have Royston-Parmar models been applied for prognostic modeling in health research?”. The scoping review protocol was registered with Open Science Framework (https://osf.io/r3232/).

### Search strategy

A search strategy consisting of three approaches—indexed databases, gray literature, and manual searches—was undertaken (specific details provided in Additional file 1: Appendix 1). The first approach was a comprehensive search of published literature indexed in six indexed, electronic databases (MEDLINE, EMBASE, CINAHL, Scopus, Web of Science, and the Cochrane Library). With the guidance of a librarian, a search string consisting of subject headings and keywords related to the research question was created. Specifically, subject headings for survival analysis and health research (e.g., epidemiology, medicine, public health, health services research) were identified and combined with keywords for the Royston-Parmar model, the model’s features (e.g., restricted cubic splines), and statistical software commands used for estimating Royston-Parmar models (e.g., Stata *sptm2* command). Where appropriate, plurality, alternative spelling, and synonyms (e.g., flexible parametric model) were used. The search string was tailored to each electronic database’s syntax.

The second approach searched the gray literature indexed in web sources with limits that restricted the search to relevant hits. Search strings related to the Royston-Parmar model and flexible parametric survival models applied in Google Scholar and limited to the first 200 hits. Additionally, web searches using the Google search engine were performed and limited to the first 30 search results—one for the Royston-Parmar model and a second for flexible parametric survival models. If the result was a web page instead of a publication, the result was explored for publications and/or publication lists. The cutoff limit was different between the two searches because the relevancy of the hits varied by search type. All Google-related searches were conducted on October 26, 2016.

The third approach was manual searches for potentially missing documents. A citation search was used, which looks for a key article in the reference list of documents. In this review, a citation search using PubMed was conducted using selected foundational articles relating to the creation and methodological development of the Royston-Parmar model [14, 18–20, 22, 23, 26–29] as the key articles. Finally, all articles listed on the personal websites of selected primary authors that published on the creation and development of the Royston-Parmar model were also searched (i.e., Paul C. Lambert, Patrick Royston, and Mahesh K.B. Parmar).

Each approach was restricted to studies involving human subjects, disseminated in the English language, and published from 2001 to 2016. While the seminal article describing the Royston-Parmar model was published in 2002 [14], a companion technical article describing the estimation of the Royston-Parmar model with Stata was published earlier in 2001 [26].

### Study inclusion criteria

This scoping review focused on documents that described the application of the Royston-Parmar model in the development and/or validation of a prognostic model for a health-related outcome. This requirement led to the exclusion of:

i. Systematic reviews;
ii. Methodology articles where the main aim was to describe a methodological development related to the Royston-Parmar model, including articles in which the methodology was demonstrated using real-world or simulated data;
iii. Technical reports describing how to model data with the Royston-Parmar model within statistical software (e.g., Stata);
iv. Associational studies and description of trend studies where the main aim was to examine the association between risk (/protective) factor(s) and time-to-event outcomes, rather than for risk prediction.

Articles were also excluded if the outcome was unrelated to clinical health, population health, or the social determinants of health. Articles with only an abstract (e.g., conference proceedings) were excluded because of the lack of reported methodological detailed regarding the Royston-Parmar model. Both univariable and multivariable prognostic models were considered.

**Fig. 1:**
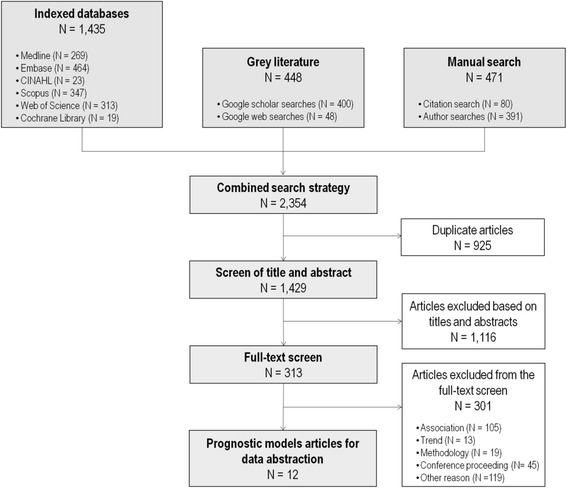
Flow diagram of studies included for the scoping review

After combining all documents and removing duplicates using the Mendeley reference management software, 1429 articles were eligible for inclusion (Fig. 1). Exclusion criteria were applied at two levels of assessment: a first screening based on the title and abstract (1116 articles excluded) followed by a second screening of the full text for remaining documents (301 excluded). Both assessments were conducted independently by two reviewers (KK and RN) and logged into a standardized, piloted relevance form. Disagreements between the two reviewers were resolved through discussion against the inclusion criteria. After the assessments, 12 articles were eligible for abstraction [30–41].

### Data abstraction and synthesis

Data were abstracted using a standardized, piloted abstraction form. Data were abstracted by one reviewer and verified by the second reviewer; disagreements were resolved through discussion. The data were abstracted and synthesized according to three themes—study characteristics, specifications of the Royston-Parmar model, and factors corresponding to the application of the Royston-Parmar model to prognostic modeling of health-related outcomes—described briefly below. If the article described more than one prognostic model constructed using the Royston-Parmar model, details from each model were abstracted. The prognostic model described in most detail was identified as the main prognostic model, and its characteristics are presented with the other models presented as sensitivity analyses in relation to the main model.

1. Study characteristics
  - *General study characteristics*, such as author, year of publication, and subject area (e.g., cancer, cardiovascular, aging); and
  - *Study methods*, such as cohort details (e.g., study location, study setting, study size), prognostic factors, and outcomes.

2. Royston-Parmar model specifications, such as number of knots, placement of knots, sensitivity analysis of the number and placement of knots, software used, and model extensions (e.g., relative survival, competing risk);
3. Application of the Royston-Parmar model to prognostic models of health-related outcomes
  - *Development and validation details*, such as measures of overall performance (e.g., *R*^2^), discrimination (e.g., Harrell’s C statistic), calibration (e.g., Hosmer-Lemeshow goodness of fit), and validation (e.g., internal, external validation);
  - *Results reported from the Royston-Parmar model*, including baseline hazard functions (equation or figure), hazard rates, survival rates, and cure proportions;
  - *Sensitivity analysis and comparisons to other survival analysis methods* including goodness of fit measures (e.g., Akaike information criterion (AIC))*;* and
  - *The rationale reported for using a Royston-Parmar model*, including its benefits and limitations.

## Results

There were 12 studies that applied the Royston-Parmar model for the development and/or validation of a prognostic model for health research [30–41]. Ten studies used the Royston-Parmar model as the main survival analysis method for their prognostic model [30–35, 37–39, 41]; the other two studies used it to complement their Cox PH-based prognostic model approach [36, 40]. In these two studies, the Royston-Parmar model was used in one instance to depict the unadjusted hazard function [40] and in the other to show the adjusted survival curve [36]. Two of the studies had developers of the Royston-Parmar model as co-authors [34, 41].

### Study characteristics

Table 1 summarizes the study characteristics. All studies were published from 2012 onwards with the most published in 2016 (*n* = 4). The studies spanned eight countries with the largest number originating from the UK (*n* = 3), the country from where the model originated. The prognostic models were developed in six subject areas, with half the studies related to cancer (*n* = 6). Most prognostic models used either administrative data (*n* = 7) or clinical data (*n* = 3), with the study setting as specific as a hospital (*n* = 4) to as broad as a country (*n* = 3) and the sample sizes ranging from 185 to 870,878 individuals. The most common event measured was mortality (*n* = 10) and maximum follow-up time ranged from 0.5 to 21 years. All studies except one used calendar time as the timescale; the other used age.

**Table 1:** Study characteristics of prognostic model studies using flexible parametric survival models

Author (year)	Country	Topic area of research	Data source	Study setting	Sample size	Maximum follow-up time	Timescale	Event/outcome	Number of events
Andersson et al. (2014) [34]	Sweden	Cancer	Administrative data	Country	5850	15 years	Calendar time	Mortality	1951
Baade et al. (2015) [33]	Australia	Cancer	Administrative data	Country	870,878	21 years	Calendar time	Mortality	261,720
Baade et al. (2015) [41]	Australia	Cancer	Administrative data	Secondary care	28,654	16 years	Calendar time	Mortality	5469
Castillo et al. (2013) [38]	United States of America	Cancer	Administrative data	Primary care	2284	12 years	Calendar time	Mortality	1210
Csordas et al. (2016) [37]	Switzerland	Cardiovascular	Clinical data	Hospital	185	222 days	Calendar time	Mortality	17
Eyre et al. (2012) [40]	United Kingdom	Infectious disease	Administrative data	Hospital	1678	4.6 years	Calendar time	*Clostridium difficile* infection recurrence	363
Fox et al. (2014) [32]	United Kingdom	Cancer	Clinical data	Hospital	2918	Not stated	Calendar time	Mortality	Not stated
Li et al. (2016) [39]	United Kingdom	Organ transplant	Administrative data	Secondary care	12,307	10 years	Calendar time	Mortality	1503
Miladinovic et al. (2012) [30]	United States of America	Aging	Medical records	Hospice	590	371 days	Calendar time	Mortality	590
Myklebust et al.(2016) [31]	Norway	Cancer	Administrative data	Country	805,365	15 years	Calendar time	Mortality	Not stated
Ramezani Tehrani et al. (2016) [35]	Iran	Reproductive/perinatal	Population survey	Community	1015	12.3 years	Age	Menopause	277
Sanchis et al. (2014) [36]	Spain	Cardiovascular	Clinical data	Hospital	342	34 months	Calendar time	Mortality	74

### Royston-Parmar model specifications

Table 2 summarizes the Royston-Parmar model specifications of included studies. Ten of the 12 studies used the Royston-Parmar model as the basis for their prognostic model, 4 of which used the model in a relative survival model, and 2 of which incorporated cure models; all relative survival models were for cancer studies. Only one of the 12 studies focused on the improvement in prognosis when biomarkers were added to the model [37]. Eight of the 12 studies reported the number of knots. Studies were more likely to specify the number of knots in terms of degrees of freedom (*n* = 6) as opposed to the number of knots (*n* = 2). The number of interior knots used in the eight studies ranged from 1 to 6. Only three of these studies described the placement of knots, all of which spaced the knots evenly across the distribution of uncensored log event times. Five of the 12 studies reported conducting sensitivity analyses to check the number and/or placement of knots using AIC or Bayesian information criterion (BIC). Interestingly, four studies incorporated sensitivity analyses that looked at the fit of the restricted cubic spline on the PO and/or probit scales. One study compared the fit of the restricted cubic spline on the PO and PH scales and found that the PO scale led to the best fit. Three studies compared all three scales, and two found the probit scale provided the best fit while the other found the PH scale provided the best fit. Nine of the 12 studies used the PH scale for their Royston-Parmar model. No studies specified whether they orthogonalized their bases functions. Eleven studies reported conducting their analysis in Stata, five of which reported using the updated command for Royston-Parmar models, *stpm2*.

**Table 2:** Royston-Parmar model specifications

Author (year)	Country	Reason for flexible parametric survival model	Relative survival model	Number of interior knots (i.e., df-1)	Expressed as knots or degrees of freedom?	Placement of knots	Sensitivity analysis for knots	Scale used	Software (command)	Candidate variables	Variable selection strategy	Variables in final model
Andersson et al. (2014) [34]	Sweden	Prognostic model	Yes, cure model	5	Degrees of freedom	Evenly spaced	Not stated	PH	Stata (stpm2)	4	Not stated	4
Baade et al. (2015) [33]	Australia	Prognostic model	Yes	6	Degrees of freedom	Not stated	Not stated	PH	Stata (stpm2)	3	Not stated	3
Baade et al. (2015) [41]	Australia	Prognostic model	No	2	Degrees of freedom	Not stated	Compared models with varying number of knots on the PH, PO, and probit scales using BIC	Probit	Stata (stpm2)	9	Backwards selection	6
Castillo et al. (2013) [38]	United States of America	Prognostic model	Yes, cure model	Not stated	N/A	N/A	Not stated	PH	Stata (not stated)	5	Not stated	5
Csordas et al. (2016) [37]	Switzerland	Prognostic model	No	1	Degrees of freedom	Not stated	Not stated	PH	Stata (not stated)	4	Not stated	2
Eyre et al. (2012) [40]	United Kingdom	Complement Cox PH prognostic model	No	Not stated	N/A	N/A	Models compared with AIC; the types of models compared are not described	PH	Stata (not stated)	Royston-Parmar model not used for model	Royston-Parmar model not used for model	Royston-Parmar model not used for model
Fox et al. (2014) [32]	United Kingdom	Prognostic model	No	Not stated	N/A	N/A	Not stated	PH	Stata (stpm2)	10	Backwards selection	10
Li et al. (2016) [39]	United Kingdom	Prognostic model	No	2	Knots	Not stated	Compared models with 0 to 4 knots on the PH, PO, and probit scales using AIC	PH	Stata (stpm2)	17	Backwards selection	6
Miladinovic et al. (2012) [30]	United States of America	Prognostic model	No	1	Knots	Evenly spaced	Compared models with 0 to 5 knots on the PH, PO, and probit scales using AIC, BIC, and *R*^2^	Probit	Stata (not stated)	1	Not stated	1
Myklebust et al.(2016) [31]	Norway	Prognostic model	Yes	4	Degrees of freedom	Evenly spaced	Not stated	PH	Not stated	3	Not stated	3
Ramezani Tehrani et al. (2016) [35]	Iran	Prognostic model	No	1	Degrees of freedom	Not stated	Compared model with 2 knots on PH scale versus model 1 knot on PO scale using AIC	PO	Stata (not stated)	4	Forward selection	2
Sanchis et al. (2014) [36]	Spain	Complement Cox PH prognostic model	No	Not stated	N/A	N/A	Not stated	PH	Stata (not stated)	Royston-Parmar model not used for model	Royston-Parmar model not used for model	Royston-Parmar model not used for model

### Application of the Royston-Parmar model to prognostic models of health-related outcomes

Of the ten studies that based their prognostic model on a Royston-Parmar model, six conducted internal validation (two used split-sample validation [30, 39], three used cross-validation [32, 35, 41], and one used bootstrap validation [38]), and two others conducted geographical external validation [32, 37]. Overall measures of performance to account for the variation explained using *R*^2^ was reported in three studies [30, 35, 41]. Discrimination—the ability to distinguish between individuals with and without the event—was reported in five studies [30, 32, 37, 39, 41]. The most commonly reported measure was Harrell’s c statistic (*n* = 4) [32, 37, 39, 41], a discrimination measure that represents the probability an individual is correctly identified as having a longer survival time than another random subject while considering censoring [42]. One study reported the Royston and Sauerbrei’s D statistic [41]—the separation between the survival distributions of two groups [43]—and one reported discrimination (Yates) slopes [30]—the separation in average predictions between subjects with and without the event [44]. Calibration—the agreement between observed outcomes and predictions—was reported in six studies with five studies comparing observed results with predicted results using Kaplan-Meier plots [30, 32, 37, 39, 41]. One study compared predicted estimates to life table estimates [34], another study conducted Hosmer-Lemeshow tests [37], and a third study calculated scaled Brier scores [30]. In the one study that examined the prognostic value of biomarkers [37], category-free net reclassification improvement and integrated discrimination improvement were used, which are two measures that examine how individuals are reclassified based on the addition of a new prognostic factor. All measures of performance used in the studies were the same as the measures of performance used in other survival analysis models (i.e., they did not need to be adapted to the flexible parametric survival model).

Across studies, 15 different estimates were reported from the prognostic models (Table 3). The most common estimates reported were survival-related estimates (*n* = 9) (i.e., survival, relative survival, net survival) and hazard rate-related estimates (*n* = 5) (i.e., hazard rate, excess hazard rate). Only three studies reported information related to a baseline function: two provided a figure of a baseline function (one was a survival curve [30], the other was a hazard function [32]), and the third provided the coefficients for the derived variables of the restricted cubic spline [39]. No studies provided enough details for their restricted cubic spline to permit the reader to use the model for full prediction or to validate the model. Nine of the ten Royston-Parmar prognostic models reported at least one type of summary estimate for prediction: prediction table (*n* = 4) [31, 33, 35, 41], survival curve (*n* = 3) [30, 39, 41], score chart (*n* = 2) [32, 38], or a figure (*n* = 1) [34]. The Cox proportionality assumption was considered in nine studies [30, 31, 33–35, 37–41]. Four of these studies tested for non-proportionality explicitly using either log-log plots (*n* = 1) [39], test for proportionality using Schoenfeld residuals (*n* = 1) [30], or interactions with time (*n* = 2) [38, 41]. Of the other five studies, one study assumed non-proportional hazards (*n* = 1), while the other four did not report formal testing (*n* = 4). Three of the nine studies included interaction terms to account for non-proportional hazards [31, 32, 37], two of which used a second restricted cubic splines to model the interaction (as opposed to a linear interaction term) [31, 32]; this restricted cubic spline is different than the spline used to model the baseline cumulative hazard. Of the two studies that used a restricted cubic spline to model the interaction, neither study reported enough details to allow the reader to reconstruct this other restricted cubic spline. Three other studies had violations of the proportionality assumptions [38, 39, 41], but they were not considered in the final prognostic model because the estimates and/or the model fit did not change when non-proportionality was considered in the model.

**Table 3:** Parameters estimated using flexible parametric survival models

Author (year)	Cumulative hazard	Cumulative incidence	Cure difference	Cure proportion	Hazard rate-related estimate	Hazard ratios	Loss of life expectancy	Median survival time differences	Median survival time of the uncured	Relative survival ratio differences	Remaining life expectancy	Survival-related estimate
Andersson et al. (2014) [34]	–	–	Yes	Yes	–	–	–	Yes	Yes	Yes	–	Relative survival
Baade et al. (2015) [33]	–	–	–	–	–	–	Yes	–	–	–	Yes	Relative survival
Baade et al. (2015) [41]	–	–	–	–	–	–	–	–	–	–	–	Survival
Castillo et al. (2013) [38]	–	Yes	–	–	Excess hazard rate	Yes	–	–	–	–	–	Relative survival
Csordas et al. (2016) [37]	Yes	–	–	–	Hazard rate	Yes	–	–	–	–	–	Survival
Eyre et al. (2012) [40]	Yes	–	–	–	Hazard rate	–	–	–	–	–	–	–
Fox et al. (2014) [32]	–	–	–	–	Hazard rate	Yes	–	–	–	–	–	Survival
Li et al. (2016) [39]	–	–	–	–	Hazard rate	Yes	–	–	–	–	–	Survival
Miladinovic et al. (2012) [30]	–	–	–	–	–	–	–	–	–	–	–	Survival
Myklebust et al.(2016) [31]	–	–	–	–	–	–	–	–	–	–	–	Net survival
Ramezani Tehrani et al. (2016) [35]	–	Yes	–	–	–	–	–	–	–	–	–	–
Sanchis et al. (2014) [36]	–	–	–	–	–	–	–	–	–	–	–	Survival

For studies that included other survival analysis models (including the two studies that did not use flexible parametric survival models in their prognostic model), only the parameters estimated directly using flexible parametric survival models are reported

In four studies, the Royston-Parmar model was compared to another survival model (one to a Cox model [30], two to a Weibull model [35, 39], and one to two non-parametric approaches for estimating net survival, the period and hybrid approaches [31]).The two studies comparing the Royston-Parmar model to a Weibull model reported a better fit of the baseline hazard with the Royston-Parmar model, while the other two studies reported improved prediction accuracy using the flexible survival approach versus the Cox model and non-parametric approaches. Nine studies provided at least one benefit for using the Royston-Parmar model (Table 4) with the main reasons being that this model improved model accuracy (*n* = 5) [30, 31, 34, 35, 41] and that they allowed the baseline function to be modeled in a flexible manner (*n* = 4) [30, 32, 39, 41]. Two studies mentioned limitations of the Royston-Parmar model, which were the baseline hazard could be overfitted [32], and the difficulties of interpreting a model with multiple time-dependent effects because the hazard ratios are dependent on more than one covariate [41].

**Table 4:** Features of flexible parametric survival models reported by the prognostic model studies

Features of flexible parametric survival models	*n*	(%)
Benefits		
Additional insights of prognostic factors versus other survival analysis models	1	(8.3)
Compute additional parameter estimates versus other survival analysis models	1	(8.3)
Extrapolation using the linear tail of the restricted cubic spline	2	(16.7)
Flexibly fit (/model) the baseline function	4	(33.3)
Improved model accuracy	5	(41.7)
Model time-dependent effects	1	(8.3)
Validation in other settings	2	(16.7)
No reported benefits	3	(25.0)
Limitations		
Overfitting	1	(8.3)
Difficult to interpret models with more than one time-dependent effect	1	(8.3)
No reported limitations	10	(83.3)

## Discussion

Our scoping review revealed that the application of the Royston-Parmar model for prognostic modeling is not commonly used. As of October 2016, only ten prognostic models were built using a Royston-Parmar model, even though this model was introduced in 2002 [14]. A key advantage of Royston-Parmar model is the ability to model the baseline log cumulative hazard function flexibly with a restricted cubic spline so complex functions can be fit and used for continuous, mathematical risk prediction for a variety of measures like hazard rates, differences in survival probabilities, standardized survival functions, and cure proportions [14]. In two instances, the developers of a Cox-based prognostic model utilized the Royston-Parmar to complement their Cox-based prognostic model; however, no reasons were provided as to why the Cox PH model was selected over the Royston-Parmar model.

The most common benefit described by the studies was that the Royston-Parmar model improved model accuracy (*n* = 5). Two studies referenced this benefit while the other three studies that made this claim provided evidence from their data by comparing the calibration of the Royston-Parmar model to other models (e.g., Cox PH model), two of which used apparent validation while the other used internal validation. While this potential benefit is based on a select, few studies, if the Royston-Parmar model does generally improve model calibration versus other survival models, this further advocates for the development of prognostic models using the Royston-Parmar model, especially because the model can easily estimate absolute measures of effect. Reporting comparisons of performance measures (i.e., calibration and discrimination) between the Royston-Parmar model and traditional models (e.g., Cox PH model) during model development in future studies would clarify this benefit. One study did not mention model accuracy as a benefit but did find better discrimination in the Royston-Parmar prognostic model versus the Cox PH prognostic model during external validation [32]. More research is needed to understand how the predictive performance of the Royston-Parmar model compares to other survival models during validation, in particular for external validation which assesses the model’s generalizability.

The most surprising finding from this scoping review was the lack of complete reporting around the restricted cubic spline. The second most common strength of the Royston-Parmar model as found in this review was that it permitted the baseline function to be modeled in a more flexible manner compared to parametric PH survival models, which constrain the baseline function to specified distributions. This benefit implies that researchers were aware that prognostic models could provide continuous risk prediction. Despite this benefit, no study reported enough details to allow others to reconstruct the baseline function for future use. Two pieces of information related to the restricted cubic spline are required to mathematically predict risks from the Royston-Parmar model—the derived variables and their coefficients. None of the studies provided enough information to determine the derived variables, which can only be calculated if the values of the knot positions are provided explicitly. Only three of the studies mentioned the placement of the knots, all of which mentioned the interior knots were evenly spaced. However, this is not sufficient information to calculate the exact placement of the knots because the knot positions are dependent on the distribution of the uncensored log event times, which is data-specific. The coefficients of the derived variables are also required, yet only one study published them. There was also a lack of reporting as to whether orthogonalization was performed, which is not necessary for the post-estimation, but provides further details as to how the restricted cubic spline was specified. Possible reasons as to why the complete information was not published include lack of available space in the article or proprietary reasons. Unfortunately, by not reporting all of the model parameter estimates such as the restricted cubic spline used to fit the baseline log cumulative hazard function, not only is the ability for complete risk prediction prevented, but it stops other researchers from applying and validating the model in other settings. Validation was raised as a benefit by two of the studies, yet ironically, not enough information was provided in either study to permit validation of their complete prognostic model. However, nine of the studies did provide simplified methods for prediction. Based on the scoping review findings that construction of the baseline cumulative hazard functions were different between studies and that the reporting of the baseline function varied across studies, we present model-building considerations for the baseline cumulative hazard function and reporting suggestions for the Royston-Parmar model to help address concerns about overfitting and to increase the transparency of how this model is used in practice.

### Model building considerations for the baseline cumulative hazard function

The main two considerations for modeling the baseline cumulative hazard function with a restricted cubic spline are the number of knots and their placement. While previous research suggests that the baseline cumulative hazard function of the Royston-Parmar model is generally robust to the number and placement of knots [14, 18, 19], we believe it is important to still examine the number of knots and their placement to ensure the specified restricted cubic spline fits the model well and to understand how robust the model is to different parameterizations of the restricted cubic spline. In terms of the number of knots, previous research on the general use of restricted cubic splines suggests that splines with more than five knots are seldom required [45]. Based on this, we recommend that restricted cubic splines ranging from two to six knots (i.e., zero to four interior knots) should be examined, but up to eight knots (i.e., six interior knots) may be needed as evidenced by some studies included in this review. The two-knot model (i.e., zero interior knots) is equivalent to a parametric survival model with a Weibull distribution.

For a given number of knots, Royston and Parmar originally suggested placing the boundary knots at the smallest and largest uncensored log survival times and then to place the internal knots such that they divide log time into equal percentiles [14]. This knot placement strategy is based on previous recommendations for knot placement when generally using restricted cubic splines [20]. For example, a restricted cubic spline with three internal knots would have the internal knots positioned at the 25th, 50th, and 75th percentiles. One simulation study found that using this strategy, if a sufficient number of knots are used in a Royston-Parmar model, the fitted hazard functions are similar to the true function and that the estimated relative effects (i.e., HRs) are insensitive to the correct specification of the baseline function [20]. Another strategy suggested by Royston is that for a given number of knots, the placement of the knots can be randomized many times, and the model in which the position of knots results in the best model fit as measured with an information criterion (e.g., AIC, BIC, both) is used [46]. However, selecting knot position in this manner may result in overfitting, and no studies have examined this strategy on parameter estimation. A third strategy is that if the baseline function has been well-characterized in the literature (e.g., the model has been previously validated), the number of knots and their placement is based on this shape. Like the randomized knot strategy, no studies have looked at the effectiveness of this strategy.

Regardless of the strategy used, as our scoping review found, the fitted baseline cumulative hazard functions from different models can be compared with a measure of information criterion (e.g., AIC, BIC, both) or a plot of the baseline function. Plots can help identify issues of underfitting and overfitting. If the information criterion estimates are similar between models, we recommend selecting the model with fewer knots as using additional knots will improve discrimination and/or calibration during model development, but may lead to overfitting during model validation. Model fit can also be examined across different scales (i.e., PH, PO, probit), but we recommend the choice be guided by how the model will be used and interpreted. If the PH scale is chosen, the robustness of the predictor regression coefficients can be compared with the coefficients from a Cox PH model. There are many options available to implement the Royston-Parmar model. The most common option is the *sptm2* command in Stata, which also includes easy-to-use post-estimation tools to measure absolute measures of effect such as hazard rates, cumulative hazard, population-averaged survival functions, and cure proportion. In R, the *flexsurv* and *Rstpm2* packages are available for modeling, with *Rstpm2* having the ability to model penalized splines. In SAS 14.1, *PROC ICPHREG* in SAS is an alternative as well. Lastly, general model building strategies for prediction models (e.g., missing data, variable selection, validation) should also be considered when using the Royston-Parmar model [47].

### Reporting suggestions for the baseline cumulative hazard function

Not only is it important to correctly specify the baseline cumulative hazard function, it is also important to report details about the baseline function. Reporting all details about the baseline function will improve the transparency of the final model and permit other people to use and/or validate the model. The “Methods” section should describe in adequate detail the process of specifying the restricted cubic spline, including any strategies used to test the robustness of the restricted cubic spline such as whether the number of knots and/or the knot placements were varied, the types of scales examined (e.g., PH, PO, probit), and the model selection strategies used (e.g., information criterion and/or plots). Details about the restricted cubic spline used to model the final baseline cumulative hazard function should be reported including the number of knots and the exact placements of the knots on the log timescale (i.e., their values)—and not just their percentiles—so the derived variables can be determined. The coefficients of the derived variables should also be reported. If the derived variable functions were orthogonalized, this should also be reported. Ideally, this information is provided in the text of the article, but alternatives include a supplementary appendix or a statement that the information can be provided upon contact with the researchers. The code used to model the data should also be provided to increase transparency. The suggestions provided for reporting the baseline cumulative hazard function here should be used in conjunction with general reporting guidelines (e.g., TRIPOD, REMARK) for prognostic models so that all aspects of model development and/or validation (e.g., sources of data, participants, candidate predictors, missing data, statistical analysis methods, model development, model validation) are transparent [3, 48].

### Time dependency

A feature of the Royston-Parmar model highlighted by the creators, but was only mentioned once in our review, was that the Royston-Parmar model permits flexible modeling of a time-dependent effect by including a second restricted cubic spline. Consideration of time dependency is especially important in studies with long follow-up time where violations of the proportionality assumption are more likely to occur. Two of the studies modeled time dependency using a second restricted cubic spline in their prognostic model. However, sufficient details for both the baseline and the time-dependent restricted cubic splines must be provided for time-dependent prediction. If time dependency is also modeled using restricted cubic splines, its details should be reported in a similar manner as the restricted cubic spline used to model the baseline cumulative hazard function. One limitation of using restricted cubic splines for time-dependent effects is that when there is more than one time-dependent effect, interpreting relative effects is difficult (e.g., HR); however, absolute measures of effect are more important for prognostic models, so the increased precision from modeling the time-dependent effects with restricted cubic splines may outweigh the interpretability of the relative effects. A solution to this limitation has been proposed where another flexible parametric survival model is used where the baseline log hazard function is modeled as a restricted cubic spline (as opposed to the log *cumulative* hazard function) [49]. While modeling the baseline hazard function as a restricted cubic spline is not a new idea [50, 51], this model is more computationally intensive than the Royston-Parmar model [49].

### Limitations

To our knowledge, there are no subject headings in the indexed databases for Royston-Parmar models, so our search strategy relied on keyword searches. However, an inherent limitation of keyword searches is that it is limited to the title and abstract. In particular, this scoping review focused on a survival analysis method that is less likely to be described in the title and abstract compared to non-methodological content (e.g., interventions for disease prevention), which would reduce the accuracy of keyword searching. Thus, the search strategy may have missed articles resulting in an underestimation of the number of studies that applied the Royston-Parmar model for prognostic modeling. However, the Royston-Parmar model is a relatively novel survival analysis method, so it is more likely to be mentioned in an abstract compared to a traditional method like the Cox PH model. As well, three approaches were used in the search strategy to be comprehensive, including citation searches of all articles that were significant to the creation and methodological development of the Royston-Parmar model [14, 18–20, 22, 23, 26–29]. Despite this wide-ranging search strategy, there was at least one instance where an article was missed by our search strategy [52]. In this instance, the abstract had no keywords listed in the search strategy, and the citation search did not identify the article even though the paper cited the original paper by Royston and Parmar.

## Conclusions

The feature of the Royston-Parmar model to flexibly model the baseline log cumulative hazard function with a restricted cubic spline for continuous mathematical risk prediction of absolute measures of effect (e.g., hazard rates, survival function) makes it an ideal survival analysis method for prognostic modeling. However, this scoping review shows that this model has only been used a handful of times for prognostic modeling in a health context. The scoping review also showed that key pieces of information required to reconstruct the baseline restricted cubic spline are rarely reported (i.e., the exact placement of the knots across the uncensored log event times and the coefficients of the derived variables). We have provided model building considerations and reporting suggestions for prognostic models built using the Royston-Parmar model to address model overfitting, enhance transparency in model development, and aid model validation and adaptation by others.

## Additional file

Additional file 1: Appendix 1. Search string syntax, by electronic database and search approach. (DOCX 17 kb)

## Abbreviations

AIC: Akaike information criterion
BIC: Bayesian information criterion
HR: Hazard ratio
KK: Kathy Kornas
PH: Proportional hazards
PO: Proportional odds
RN: Ryan Ng
