# Supplementary material for: The current application of the Royston-Parmar model for prognostic modeling in health research: a scoping review
Source: Diagn Progn Res. 2018 Feb 7;2:4. doi: 10.1186/s41512-018-0026-5 (PMC6460777; doi:10.1186/s41512-018-0026-5)
Supplement: Supplementary file 1 — Appendix 1. Search string syntax, by electronic database and search approach. (DOCX 17 kb) [file 41512_2018_26_MOESM1_ESM.docx]

# Appendix 1: Search string syntax, by electronic database and search approach

## Medline

1. exp Medicine/

2. exp Health/

3. exp Epidemiology/

4. exp Health Services Research/

5. exp Public Health/

6. exp Preventive Medicine/

7. exp Survival Analysis/

8. exp Survival Rate/

9. exp Models, Statistical/

10. (flex* parametr* adj4 (model* or anal*)).ti,ab.

11. ((Royston adj2 Parmar) or Royston-Parmar).ti,ab.

12. (stpm* or rstpm2 or strsrcs or flexsurv or proc iphreg).ti,ab.

13. ((restrict* cubic or natur* cubic) adj2 spline*).ti,ab.

14. (splin* adj2 (model* or anal*)).ti,ab.

15. knot*.ti,ab.

16. (smooth* adj2 (funct* or estimat*)).ti,ab.

17. (hazard* or hr* or surviv* or "cumulative incidence" or kaplan or km or "k-m").ti,ab.

18. 12 or 13 or 14 or 15 or 16

19. 17 and 18

20. 7 or 8 or 9

21. 19 and 20

22. 10 or 11

23. 1 or 2 or 3 or 4 or 5 or 6

24. 21 and 23

25. 22 or 24

26. limit 25 to (human and yr="2001 -Current" and English language)

## Embase

1. exphealth/

2. exp medicine/

3. exp epidemiology/

4. exp health services research/

5. exp public health/

6. exp preventive medicine/

7. exp statistical model/

8. exp survival analysis/

9. exp proportional hazards model/

10. exp hazard ratio/

11. exp survival/

12. (flex* parametr* adj4 (model* or anal*)).ti,ab.

13. ((Royston adj2 Parmar) or Royston-Parmar).ti,ab.

14. (stpm* or rstpm2 or strsrcs or flexsurv or proc iphreg).ti,ab.

15. ((restrict* cubic or natur* cubic) adj2 spline*).ti,ab.

16. (splin* adj2 (model* or anal*)).ti,ab.

17. knot*.ti,ab.

18. (smooth* adj2 (funct* or estimat*)).ti,ab.

19. (hazard* or hr* or surviv* or "cumulative incidence" or kaplan or km or "k-m").ti,ab.

20. 14 or 15 or 16 or 17 or 18

21. 19 and 20

22. 7 or 8 or 9 or 10 or 11

23. 21 and 22

24. 12 or 13

25. 1 or 2 or 3 or 4 or 5 or 6

26. 23 and 25

27. 24 or 26

28. limit 27 to (human and yr="2001 -Current")

## CINAHL

S25 S22 OR S24 Limiters - Published Date: 20110101-20171231; Human; English

S24 S21 AND S23

S23 S1 OR S2 OR S3 OR S4 OR S5 OR S6 OR S7

S22 S10 OR S11

S21 S19 AND S20

S20 S8 OR S9

S19 S17 AND S18

S18 S12 OR 13 OR S14 OR S15 OR S16

S17 hazard* or hr* or surviv* or "cumulative incidence" or kaplan or km or k-m

S16 (smooth* n1 (funct* or estimat*))

S15 knot*

S14 (splin* n1 (model* or anal*))

S13 ((restrict* cubic or natur* cubic) n1 spline*)

S12 (stpm* or rstpm2 or strsrcs or flexsurv or proc iphreg)

S11 ((Royston n1 Parmar) or Royston-Parmar)

S10 flex* parametr* n3 (model* or anal*)

S9 (MH "Models, Statistical")

S8 (MH "Survival Analysis+")

S7 (MH "Preventive Health Care+")

S6 (MH "Public Health+")

S5 (MH "Health Services Research+")

S4 (MH "Epidemiology+")

S3 (MH "Medicine+")

S2 (MH "Disease+")

S1 (MH "Health+")

## Scopus

TITLE-ABS-KEY ( ( {flexible parametric}W/3( model*ORanal* ) )OR( {flexibly parametric}W/3( model*ORanal* ) ) )ORTITLE-ABS ( {Royston-Parmar}OR( roystonW/1parmar )OR( rstpm2ORstrsrcsORflexsurvOR{proc iphreg}ORstpm*OR( {restricted cubic}PRE/0splin* )OR( {natural cubic}PRE/0splin* )OR( knot* )OR( smooth*PRE/1( funct*ORestimat* ) )AND( hazard*ORhr*ORsurviv*OR{cumulative incidence}ORkaplanOR{K-M}ORkm ) ) )

AND

(PUBYEAR>2000)

AND

( EXCLUDE ( SUBJAREA ,"EART" )OREXCLUDE ( SUBJAREA ,"AGRI" )OREXCLUDE ( SUBJAREA ,"PHYS" )OREXCLUDE ( SUBJAREA ,"MATH" )OREXCLUDE ( SUBJAREA ,"ENGI" )OREXCLUDE ( SUBJAREA ,"ENVI" )OREXCLUDE ( SUBJAREA ,"DECI" )OREXCLUDE ( SUBJAREA ,"COMP" )OREXCLUDE ( SUBJAREA ,"ECON" )OREXCLUDE ( SUBJAREA ,"ARTS" )OREXCLUDE ( SUBJAREA ,"CENG" )OREXCLUDE ( SUBJAREA ,"BUSI" )OREXCLUDE ( SUBJAREA ,"MATE" )OREXCLUDE ( SUBJAREA ,"VETE" ) )

AND

AND(LIMIT-TO (LANGUAGE,"English”))

## Web of Science

# 13 (#11 OR #10) AND ((SU=Life Sciences & Biomedicine) OR (SU=Mathematics))AND LANGUAGE: (English)Indexes=SCI-EXPANDED, SSCI, A&HCI, CPCI-S, CPCI-SSH, ESCI Timespan=2001-2016

# 12 #11 OR #10

# 11 #2 OR #1

# 10 #9 AND #8

# 9 #7 OR #6 OR #5 OR #4 OR #3

# 8 TS= (hazard* or (cumulative incidence) or (surviv*) or hr or hrs or km or 'k-m' or "kaplan")

# 7 TOPIC: ((smooth* near/1 funct*) or (smooth* near/1 estimat*))

# 6 TOPIC: (knot*)

# 5 TOPIC: ((splin* near/1 model*) or (splin* near/1 anal*))

# 4 TOPIC: (((restricted cubic) near/1 splin*) or ((natural cubic) near/1 splin*))

# 3 TOPIC: (stpm* or rstpm2 or strsrcs or flexsurv or proc iphreg)

# 2 TS= ((Royston near/1 Parmar) or Royston-Parmar)

# 1 TS= ((flex* parametr* near/3 (model* or anal*)))

## Cochrane library

((flexible or flexibly) (parametric OR parametrically) (model* OR anal* OR funct*)) OR “Royston-Parmar” OR “Royston Parmar” OR “Royston and Parmar”

## Google Web Search (first 30 hits)

“flexible parametric” (hazard OR hr OR knot OR "restricted cubic spline" OR "natural cubic spline" OR hr OR smooth OR survival OR survivor OR km OR "Kaplan Meier" OR "Kaplan-Meier")

Royston Parmar (hazard OR hr OR knot OR "restricted cubic spline" OR "natural cubic spline" OR hr OR smooth OR survival OR survivor OR km OR "Kaplan Meier" OR "Kaplan-Meier")

## Google Scholar (limit to the first two hundred citations)

All the words:

- “flexible parametric”

Any of the words:

- hazard OR hr OR knot OR "restricted cubic spline" OR "natural cubic spline" OR hr OR smooth OR survival OR survivor OR km OR "Kaplan Meier" OR "Kaplan-Meier"

Limits:

- 2001-2016
- Set filter to English language only

All the words:

- Royston Parmar

Any of the words:

- hazard OR hr OR knot OR "restricted cubic spline" OR "natural cubic spline" OR hr OR smooth OR survival OR survivor OR km OR "Kaplan Meier" OR "Kaplan-Meier"

Limits:

- 2001-2016
- Set filter to English language only

## Hand search of personal web pages of the creators and developers of the flexible parametric survival model

- Paul C. Lambert: <http://www2.le.ac.uk/Members/pl4/publications>; limit to 2001 onwards
- Patrick Royston: <http://www.homepages.ucl.ac.uk/~ucakjpr/jprpaprs.pdf>; limit to 2001 onwards
- Mahesh Parmar: <https://iris.ucl.ac.uk/iris/browse/profile?upi=MKBPA56>; limit to 2001 onwards
